# Examining emotional functioning in misophonia: The role of affective instability and difficulties with emotion regulation

**DOI:** 10.1371/journal.pone.0263230

**Published:** 2022-02-11

**Authors:** Rachel E. Guetta, Clair Cassiello-Robbins, Jacqueline Trumbull, Deepika Anand, M. Zachary Rosenthal

**Affiliations:** 1 Center for Misophonia and Emotion Regulation, Department of Psychiatry and Behavioral Sciences, Duke University Medical Center, Durham, NC, United States of America; 2 Department of Psychology and Neuroscience, Duke University, Durham, NC, United States of America; University of Bologna, ITALY

## Abstract

Misophonia is a newly described condition characterized by sensory and emotional reactivity (e.g., anxiety, anger, disgust) to repetitive, pattern-based sounds (e.g., throat clearing, chewing, slurping). Individuals with misophonia report significant functional impairment and interpersonal distress. Growing research indicates ineffective coping and emotional functioning broadly (e.g., affective lability, difficulties with emotion regulation) are central to the clinical presentation and severity of misophonia. Preliminary evidence suggests an association between negative emotionality and deficits in emotion regulation in misophonia. Still, little is known about (a) the relationships among specific components of emotional functioning (e.g., emotion regulation, affective lability) with misophonia, and (b) which component(s) of misophonia (e.g., noise frequency, emotional and behavioral responses, impairment) are associated with emotional functioning. Further, despite evidence that mood and anxiety disorders co-occur with misophonia, investigation thus far has not controlled for depression and anxiety symptoms. Examination of these relationships will help inform treatment development for misophonia. The present study begins to disambiguate the relationships among affective lability, difficulties with emotion regulation, and components of misophonia. A sample of 297 participants completed questionnaires assessing misophonia, emotional functioning, depression, anxiety, and COVID-19 impact. Findings indicated that misophonia severity was positively associated with each of these constructs with small to medium effect sizes. When controlling for depression, anxiety, and COVID-19 impact, results from this preliminary study suggest that (a) difficulties with emotion regulation may be correlated with misophonia severity, and (b) misophonic responses, not number of triggers or perceived severity, are associated with difficulties with emotion regulation. Overall, these findings begin to suggest that emotion regulation is important to our understanding the risk factors and treatment targets for misophonia.

## Introduction

Misophonia is a newly described sound intolerance condition characterized by atypical sensory over-responsivity and emotional reactivity to repetitive, pattern-based sounds. These trigger sounds are typically human-produced oral or nasal noises (e.g., throat clearing, chewing, slurping), can be environmentally produced (e.g., pen clicking, clock ticking, [1, 2]), and are not attributable to aversive acoustic features such as volume or pitch [3]. The intensity, uncontrollability, and disproportionality of emotional reactions to trigger sounds is expressed across a range of affective responses (e.g., anger, anxiety, disgust; [3]). Common behavioral responses include glaring at or mimicking the person producing the sound, verbal aggression, and infrequent physical aggression [4], all of which adversely impact interpersonal functioning. Recent research suggests that distress from trigger noises may be influenced by context. For example, many people with misophonia report more intense reactions when the sound is produced by family members and loved ones, or in contexts where the noises are deemed socially inappropriate (e.g., chewing with one’s mouth open, pen-clicking in an exam setting [4, 5], highlighting the importance of context in both the perception of and response to triggers. In additional to interpersonal aggression and conflict, escape and avoidance behavior functions to prevent exposure and distress associated with misophonic cues. Such intolerance and avoidance of possible triggering contexts (i.e., family meals and restaurants, certain workplaces; [1, 4]) may maintain misophonic distress and, over time, lead to diminished capabilities with confidence and capability coping or regulating emotions.

Misophonia, as currently assessed using self-reported and clinical interview measures (e.g., [6, 7]), can be operationalized using a tripartite symptom presentation: (a) presence of bothersome sounds and related sensory cues (i.e., visual stimuli related to the sounds, such as seeing someone chewing from afar in the absence of hearing chewing); (b) heightened physiological, affective, and cognitive distress with accompanying behavioral reactions in response to one (or more) of the auditory and visual cues; and (c) associated impairment in work, academic, or social functioning. As such, emotions (conceptualized as including physiological, cognitive, and behavioral responses in order to fulfill context-dependent needs [8]); and the way in which emotions are experienced and managed are central to the clinical presentation and severity of misophonia.

Emotions are typically managed through emotion regulation: the ability to both automatically and consciously apply sets of strategies that influence emotional experience, intensity, and expression [9]. Successful emotion regulation, given the reported intensity of negative emotions in the face of aversive auditory and related stimuli, is essential to adaptive coping and management of misophonia. Chronic dysregulated emotions, particularly in the moments of being activated by triggers, can exacerbate overall impairment and subjective distress. Individuals with misophonia may attempt to disengage from the aversive stimuli and associated internal experiences via escape or avoidance. These strategies, while effective in the short-term to ease contact with the discomfort, are ineffective over time among emotional disorders broadly [10–12]. For example, dysregulated anger or disgust in response to misophonic sounds could result in repeated avoidance of interpersonal situations leading to isolation and loneliness, thereby contributing to impairment caused by misophonia. Although speculative, difficulties with emotion regulation may have an important role in the development, maintenance, and treatment of misophonia. To date, however, very little scientific research has been conducted in this area.

Preliminary evidence suggests that emotion regulation deficits mediate the relationship between trait neuroticism, negative affect, and misophonia [13]. These findings suggest that a reciprocal relationship between the tendency to frequently experience intense emotions and deficits in emotion regulation may confer risk for development and maintenance of misophonia. However, this study was preliminary, limited by small sample size, and did not control for the presence of other emotional problems that are known to co-occur with misophonia (i.e., depression, anxiety [14, 15]. As such, the relationship between emotion regulation and misophonia needs to be replicated and clarified in larger samples.

Furthermore, emotional functioning is a complex and multi-dimensional construct that includes trait features of emotional reactivity as well as those more directly related to emotion regulation. For example, affective lability (the speed, frequency, and range of changes in affective states) is an important emotional process observed in those with certain psychiatric disorders (e.g., bipolar disorder, borderline personality disorder). More generally, affective lability is a feature underlying trait neuroticism [16] in non-clinical samples and may be a vulnerability factor for psychopathology. Affective lability is a specific process that can be examined as part of a broader effort to phenotypically characterize emotional functioning in misophonia.

No studies have examined the relative contributions and differential associations of affective lability and difficulties with emotion regulation with misophonia. Determining the relationships of these emotional processes with misophonia can help clarify intervention targets for clinicians and treatment researchers. This clarification is important, as there are currently no evidence-based interventions designed for adults with misophonia, and few randomized trials have been conducted [17, 18]. Further, despite empirical evidence that mood and anxiety disorders co-occur with misophonia [6, 14, 15], investigation of misophonia thus far has not controlled for depression and anxiety symptoms. For research to elucidate factors specifically underlying misophonia, it is necessary to increase the scientific rigor by controlling for general psychopathology. In addition, no studies have dismantled whether specific components of misophonia (i.e., frequency of exposure to triggers, ineffective emotional or behavioral responses, or severity of functional impairment caused by misophonia) are differentially related to difficulties with emotional functional within this population. Determining which components may be associated with the greatest problems with emotional functioning could further efforts for treatment development (i.e., mindfulness or acceptance-based approaches for sound tolerance versus emotion regulation strategies to target ineffective behavioral responses).

This study begins to disambiguate the relationships among affective lability, difficulties with emotion regulation, and the components of misophonia. Data were collected over the first few months of COVID-19, and due to the heightened stress and presence of anxiety and depression during the pandemic [19], we account for this psychological impact in our hypotheses and data analytic approach. First, replicating and extending the finding that misophonia-related impairment is associated with emotional functioning deficits [13], we hypothesized that misophonia would be significantly associated with (a) difficulties regulating emotions in moments of distress and (b) rapid shifts in emotional state (i.e., affective lability). We further hypothesized that difficulties managing emotions may be related to severity of misophonia over and above affective lability. We expected these relationships would hold when controlling for symptoms of anxiety and depression and the impact of COVID-19. Lastly, because avoidance and escape are common in misophonia and have been more generally associated with emotion regulation difficulties across a range of psychopathology, it was hypothesized that, among the three components of misophonia, ineffective emotional and behavioral responses to triggers would be differentially associated with greater trait difficulties regulating emotions.

## Methods

### Participants

We evaluated these aims in a nonclinical sample of 297 adults recruited using the online Amazon Mechanical Turk (MTurk) platform. Inclusion criteria required participants to (a) be able to read and understand English, (b) be between 18–65 years old, and (c) have an MTurk participant approval rating of at least 99%. In line with recommended strategies when conducting research within MTurk, our study included three attention checks [20]. Attention checks were interspersed throughout the study and consisted of brief multiple-choice questions aimed to differentiate attentive from inattentive participants. Of the 384 participants who completed the study, we utilized data from the 297 participants who responded correctly to all three attention checks.

Demographic information for the final sample is displayed in Table 1. Overall, the sample predominantly identified as male, White, and non-Hispanic. The majority reported completing college or a higher level of education and had a household income over $40,000 per year.

**Table 1:** Demographics and sample characteristics.

Variable	*M (SD)*	N (%)
Age	39.13 (11.52)	
Male		156 (52.5)
Female		141 (47.5)
Race		
White		247 (83.2)
Black or African-American		21 (7.1)
Other Asian or Other Asian American (includes India, Malaysia, Pakistan, Philippines)		10 (3.4)
More Than One Racial Group		6 (2.0)
Korean or Korean American		6 (2.0)
Other		3 (1.0)
Chinese or Chinese American		2 (0.7)
Japanese or Japanese American		1 (0.3)
Native American, American Indian, Alaska Native		1 (0.3)
Ethnicity		
Hispanic		19 (6.4)
Non-Hispanic		278 (93.6)
Education Level		
Some High School		1 (0.3)
GED		10 (3.4)
High School Graduate		28 (9.4)
Bus/Tech Training Beyond High School		12 (4.0)
Some College		79 (26.6)
College Graduate		120 (40.4)
Some Graduate School		7 (2.4)
Masters Degree		35 (11.8)
Doctoral Degree		5 (1.7)
Household Income		
0 - $10,000		26 (8.8)
$10,000 - $20,000		29 (9.8)
$20,001 - $40,000		70 (23.6)
$40,001 –$65,000		63 (21.2)
$65,001 - $100,000		56 (18.9)
More than $100,000		53 (17.8)
MQ Symptom Subscale	1.30 (.87)	
MQ Emotions and Behaviors Subscale	0.93 (.61)	
MQ Severity Rating	3.63 (2.51)	
DERS_SF Total Score	35.43 (13.61)	
ALS-18 Total Score	40.63 (14.46)	
GAD-7 Total Score	4.35 (4.73)	
PHQ-9 Total Score	4.35 (5.14)	
CEFIS	6.78 (2.87)	

*Note*. MQ = Misophonia Questionnaire; DERS-SF = Difficulties with Emotion Regulation Scale Short Form; ALS-18 = Affective Lability Scale; GAD-7 = Generalized Anxiety Disorder 7-Item Scale; PHQ-9 = Patient Health Questionnaire; CEFIS = COVID-19 Exposure and Family Impact Survey.

### Procedures

The Duke Health Institutional Review Board approved study procedures and participants provided written assent before beginning the study. All participants completed a battery of self-report questionnaires related to emotional functioning, mental health symptoms, COVID-19-related stress, and misophonia. Data were collected online via REDCap and MTurk, and participants received $5.00 for completing the surveys.

### Measures

#### Misophonia Questionnaire (MQ [6])

The MQ contains 17 items across three subscales: Symptom Subscale, Emotions and Behaviors Subscale, and Impairment Subscale. The Symptom Subscale assesses frequency of bothersome noises (i.e., people eating, repetitive tapping). The Emotions and Behaviors Subscale assesses emotional (e.g., feeling anxious/distressed, becoming angry) and behavioral (e.g., escaping or avoiding situations, covering ears) responses to trigger noises. Response options for the first two subscales are rated from 0 (“not at all true”) to 4 (“always true”) and are summed for a MQ total score of 0–68. The Impairment Subscale consists of a single self-reported rating of sound sensitivity and impairment, ranging from 0 (minimal) to 15 (severe). As per Wu et al. 2014, a cut score of seven on this item indicates clinically significant misophonia symptomology. Initial validation of the MQ demonstrated good internal consistency (*α* = .86 - .89; [6]). Cronbach’s alpha in the current sample was also good for the MQ total score (*α* = .89), as well as for the Symptom Subscale (*α* = .87) and the Emotions and Behaviors Subscale (*α* = .86).

#### Difficulties in Emotion Regulation Scale–Short Form (DERS-SF [21])

The DERS-SF is an 18-item version of the widely used 36-item DERS [22]. The DERS-SF assesses six processes associated with difficulties regulating emotions when distressed: (1) Non-acceptance of emotional responses, (2) Difficulties engaging in goal directed behavior, (3) Difficulties controlling impulsive behaviors, (4) Lack of emotional awareness, (5) Limited access to emotion regulation strategies, and (6) Lack of emotional clarity. Item responses are scored from 1 (“almost never”) to 5 (“almost always”), with higher scores indicating greater difficulty with emotion regulation. The DERS-SF total score was used in the present study, is commonly used as a global measure of difficulties regulating emotions, and is the sum of the six subscales. This score ranges from 18–90, with higher scores indicating greater difficulty regulating emotions. The DERS-SF total score had high internal consistency in the validation study (*α* = .89; Kaufman et al., 2016) as well as in the current sample (*α* = .93).

#### The Affective Lability Scale– 18 item (ALS-18 [23])

This self-report measure assesses rapid shifts in affective experience and emotional expression. Each item is rated on a scale from 0 (“very uncharacteristic of me”*)* to 3 (“very characteristic of me”). Higher total scores indicate more affective lability. The measure demonstrates good internal consistency (*α* = .87; Oliver & Simmons, 2004). Chronbach’s alpha in the current sample was excellent (*α* = .97).

#### Generalized Anxiety Disorder 7-item scale (GAD-7 [24])

This brief self-report questionnaire assesses generalized anxiety. Each item is scored on a 4-point Likert scale from 0 (“not at all”) to 3 (“nearly every day”) to assess frequency of symptoms. Higher total scores reflect more severe anxiety. The GAD-7 has excellent internal consistency (α = .92) and test-retest reliability (intraclass correlation = 0.83; Spitzer et al., 2006). Chronbach’s alpha in the current sample was .92.

#### The Patient Health Questionnaire (PHQ-9 [25])

The PHQ-9 is the depression module of the larger Patient Health Questionnaire, a self-administered questionnaire that assesses each of the nine *DSM-IV* criteria for depression (i.e., feeling down, hopeless, lack of appetite). The nine items assess symptom frequency from 0 (“Not at all”) to 3 (“Nearly every day”). Higher total scores reflect higher depression severity. The PHQ-9 has excellent internal reliability (α = .89). Internal consistency was also high in the current sample (α = .89).

#### The COVID-19 Exposure and Family Impact Survey (CEFIS [26])

The CEFIS is designed to assess the impact of the COVID-19 pandemic. The CEFIS includes 25 dichotomous ratings (“yes” or “no”) of how COVID-19 may have impacted that individual and their family, including financial stress, education and work disruptions, family or individual exposure to COVID-19, hospitalizations, and deaths. In order to obtain an indicator of the overall impact of COVID-19, we summed the 25 items; higher total scores indicate more severe impacts of the pandemic. Due to the recent onset of COVID-19 at the time of this study, however, there were no psychometrically validated measures of pandemic stress. We chose to include this unpublished measure in order to control for the effects of COVID-19 on individuals’ daily functioning.

### Data analytic plan

All analyses were conducted in SPSS 26.0 [27]. First, data were screened for normality using Shapiro-Wilk tests and any data that were non-normally distributed were log transformed. To conduct this transformation a value of “1” was added to each score because the lowest possible score on these measures was zero, and the value of zero cannot be log-transformed. Preliminary exploration of the relationships among misophonia, depressive and anxiety symptoms, and COVID-19 impact was conducted using bivariate correlations. Next, a multiple linear regression was conducted to explore the relationship between misophonia symptoms and difficulties with emotion regulation and affective lability, controlling for anxiety, depression, and COVID-19 impact. In order to examine if emotional functioning predicts misophonia symptoms broadly, a z-score was computed to derive an overall misophonia severity variable (each of the three MQ subscales was z-scored and the three z-scores were summed to create a standardized composite variable reflective of overall misophonia severity). This analytic plan enabled exploration of the relationships among study variables accounting for potentially confounding variables.

Lastly, the relationships among specific components of misophonia (i.e., MQ Symptom, Emotions and Behaviors, and Impairment Scales) and emotional functioning were examined. This analysis was conducted using a multiple linear regression with significant predictors from the previous model entered in step two to determine which MQ subscale(s) accounted for emotional functioning over and above the a priori covariates (i.e., anxious and depressive symptoms, and COVID-19 impact) in step one.

## Results

The total scores for the DERS-SF, PHQ-9, GAD-7, and ALS-18 all deviated from a normal distribution (as indicated by *p* < .05 on Shapiro-Wilk tests) and were log transformed to reduce bias. Following logarithmic transformations, all variables approximated normal distributions (corrected skewness ranged from -1.78 to .34; corrected kurtosis ranged from -1.31 to 2.76; [28, 29]). Findings are presented using the transformed variables due to the *a priori* decision to log transform variables that were not normally distributed. Analyses were conducted both with and without transforming the variables and results remained the same.

### Descriptive statistics

Descriptive statistics for all measures are included in Table 1. On average, the sample obtained a mean item score of 3.63 (*SD =* 2.51) on the Impairment Subscale, suggesting that the sample reported mild sound sensitivity on average. Over a tenth of the sample (13.47%, *n* = 40) reported clinically significant misophonia symptoms that cause impairment in their everyday lives, as indicated by a score of seven or higher on the MQ Impairment Subscale [6]. In addition, the sample’s total scores on the DERS-SF, PHQ-9, and GAD-7 fell within one standard deviation of the scores reported in their respective validation studies in non-clinical samples [21, 24, 25]. The mean score on the ALS-18 was 1.80 standard deviations above that reported in the validation study [23], which used a nonclinical college-aged sample.

### Correlations between misophonia, mental health symptoms, and emotion regulation

Pearson zero-order correlations indicated there were significant, positive correlations between misophonia severity (MQ total) and (a) depressive symptoms, (b) anxiety symptoms, and (c) COVID impact. As such, we included these variables as covariates in the remaining analyses. Additional Pearson correlations were conducted to examine associations between MQ total and the two planned measures of emotional functioning. MQ total was significantly correlated with both DERS-SF and ALS-18. All correlations are displayed in Table 2.

**Table 2:** Bivariate correlations between measures of misophonia, difficulties with emotion regulation, affective lability, COVID-19 impact, anxiety, and depression.

Variable	DERS-SF	ALS-18	CEFIS	GAD-7	PHQ-9
MQ Total Score	.45**	-.16**	.30**	.43**	.42**

*Note*.

**p < .01, *N* = 265, Correlations used logarithmically transformed variables as indicated in text. MQ = Misophonia Questionnaire; DERS-SF = Difficulties with Emotion Regulation Scale Short Form; ALS-18 = Affective Lability Scale; CEFIS = COVID-19 Exposure and Family Impact Survey; GAD-7 = Generalized Anxiety Disorder 7-Item Scale; PHQ-9 = Patient Health Questionnaire.

### Relationship among components of misophonia and emotional functioning

First, we examined the relationship between emotional functioning and misophonia symptoms with a multiple linear regression (Table 3). Step 1 controlled for age and gender, as well as symptoms of depression and anxiety, and the impact of COVID-19. Step 2 included two measures of emotional functioning: the DERS-SF, measuring specific difficulties with regulating emotions when distressed, and the ALS-18, measuring strength of variability in affective experiences. The model indicated that, together, the variables in Step 2 (difficulties with emotion regulation and affective lability) accounted for a significant proportion of the variance in overall misophonia severity, even after controlling for severity of anxiety and depression symptoms and the impact of COVID-19 (*F*(7,257) = 14.45, *p* < .0001, *R*^*2*^ = .28, *R*^*2*^ change = .04). More specifically, the model indicated that the DERS-SF but not the ALS-18 evidenced a significant association with the MQ severity score (standardized β = .26, *t*(3.69), *p* < .0001).

**Table 3:** Multiple linear regressions examining emotional functioning in misophonia.

Dependent Variable		Variables	β unstd.	SE	β std	p	R^2^	R^2^
MQ composite severity	Step 1	Age	.01	.01	.04	.520	.24	.24
		Gender	.03	.18	.01	.870		
		**GAD-7**	1.43	.49	.26	.004		
		**PHQ-9**	.97	.47	.18	.040		
		**CEFIS**	.13	.05	.16	.005		
	Step 2	Age	.01	.01	.06	.280	.28	.04
		Gender	.09	.18	.03	.602		
		**GAD-7**	1.07	.48	.19	.028		
		PHQ-9	.31	.49	.06	.523		
		**CEFIS**	.13	.04	.17	.003		
		**DERS-SF**	3.96	1.07	.26	< .0001		
		ALS-18	-.29	.44	-.04	.520		
DERS-SF total	Step 1	**Age**	.00	.00	-.11	.023	.44	.44
		Gender	-.02	.01	-.08	.107		
		**GAD-7**	.09	.03	.24	.002		
		**PHQ-9**	.16	.03	.46	< .0001		
		CEFIS	.00	.00	-.04	.457		
	Step 2	Age	.00	.00	-.09	.058	.51	.07
		Gender	-.02	.01	-.07	.112		
		**GAD-7**	.07	.03	.18	.016		
		**PHQ-9**	.13	.03	.37	< .0001		
		CEFIS	.00	.00	-.06	.203		
		**MQ Emotions and Behaviors**	.10	.02	.40	< .0001		
		MQ Symptoms	-.02	.01	-.09	.097		
		MQ Impairment	.00	.00	-.07	.259		

*Note*. Variables with *p*-values less than .05 are shown in bold. MQ = Misophonia Questionnaire; GAD-7 = Generalized Anxiety Disorder 7-item scale; PHQ-9 = The Patient Health Questionnaire; CEFIS = The COVID-19 Exposure and Family Impact Survey; DERS-SF = Difficulties in Emotion Regulation Scale–Short Form; ALS-18 = Affective Lability Scale– 18 Item.

Next, we conducted a multiple linear regression to examine the specific component(s) of misophonia related to difficulties regulating emotions (Table 3), after controlling for age, gender, depression and anxiety symptoms and the impact of COVID-19 in Step 1. Only DERS-SF was included in this model, as ALS-18 was not a significant predictor in the prior model. Step 2 of the model accounted for 50% (*p* < .0001) of the variance in DERS-SF total score (*F*(8, 256) = 33.41, *p* < .0001, *R*^*2*^ = .51, *R*^*2*^ change = .07), and revealed that the Emotions and Responses Subscale but not the Symptoms or Impairment Subscales evidenced a significant association with DERS-SF total score (standardized β = .38, *t*(5.68), *p* < .0001).

## Discussion

The primary aim of this study was to examine the relationships among difficulties with emotional functioning and specific components of misophonia. A community sample was used, with 13.5% of individuals reporting moderate or higher impairment in functioning due to sound sensitivities. This result is notable, given that the sampling procedure did not selectively recruit individuals self-identifying with misophonia. The prevalence of misophonia is unknown, with previous studies estimating approximately 17% of undergraduate students reporting moderate or higher impairment caused by misophonia (e.g., [6]). Accordingly, findings from our study represent a somewhat lower estimate of the prevalence of clinically significant misophonia compared to earlier studies with college student samples.

As hypothesized in the present study, misophonia severity was correlated with anxiety, depression, COVID-19 impact, difficulty regulating emotions, and affective lability. These results are in line with previous findings suggesting misophonia may be associated with higher depression, anxiety, and difficulty regulating emotions [6, 13, 30]. This is the first study to examine the relationship of misophonia with affective instability. The association of misophonia with affective lability is consistent with earlier suggestions that misophonia may be characterized by heightened negative emotional reactivity [1]. To the best of our knowledge, this is also the first study to examine the relationships among misophonia, emotion regulation, and the impact of COVID-19. Given growing literature highlighting the relationship of COVID-19 with increased rates of depression and anxiety (e.g., [19]), which are also associated with misophonia, it is unsurprising that the impact of the ongoing pandemic is also associated with greater misophonia severity.

Additionally, results supported our hypothesis that difficulties regulating emotions would account for misophonia severity after controlling for the presence of anxiety, depression, and COVID-19 impact. One other study has noted a relationship between misophonia and difficulties regulating emotions [13]; however, this study did not control for the presence of other mental health symptoms. Thus, the results of the current study replicate and extend those findings by suggesting difficulties with emotion regulation remain significantly related to misophonia even when controlling for anxiety and depressive symptoms. Importantly, although the amount of variance in misophonia symptoms explained by difficulties with emotion regulation was statistically significant, it was much smaller than that accounted for by the impact of COVID-19 and symptoms of depression and anxiety. Nonetheless, this finding raises the question of if, and to what extent, general psychological distress may explain the relationship between misophonia and emotion regulation. One hypothesis is that individuals with misophonia may have problems regulating emotions irrespective of current anxiety, depression, or the impact of COVID-19. Responses to the DERS-SF represent more trait-like difficulties with emotion regulation, whereas the PHQ-9 and GAD-7 represent distress in the two weeks prior to assessment. As such, these findings suggest that an individual’s perception of their ability to regulate emotions broadly may be associated with misophonia above and beyond the distress they experienced close to the time of assessment. Still, because symptoms probed on the PHQ-9 and GAD-7 may also be correlated with emotion regulation, it is important to conduct research that can investigate and ultimately elucidate the roles that these constructs have in the etiology and maintenance of misophonia.

In contrast to the role of difficulties with emotion regulation, findings using a multivariate model indicated that affective lability did not account for misophonia severity when co-varying the impact of COVID-19, anxiety, and depressive symptoms. This finding points to the possibility that affective lability, though correlated with misophonia, is not as useful of an indicator of severity compared to difficulties regulating emotions. When considering this pattern of results, it is possible that the experience of strong and variable emotions is less related to misophonia symptom severity than how emotions are regulated when one is distressed. Because this is the first study to explore the relationship between affective lability and misophonia, this result should be considered preliminary until replicated. Future studies, particularly research using ecological momentary assessment and longitudinal designs, can further disambiguate the temporal relationships among affective lability and emotion dysregulation in naturalistic contexts including trigger sounds.

An additional goal of this study was to explore whether specific aspects of misophonia were differentially associated with emotional functioning difficulties. To the best of our knowledge, no study has explored whether the frequency of exposure to misophonic sounds, emotional and behavioral responses to sounds, or reported impairment due to sound sensitivity is uniquely associated with emotional functioning. Our results are congruent with the hypothesis that emotions and responses when hearing a bothersome noise, rather than the frequency of exposure to bothersome sounds, may be associated with greater difficulties regulating emotions. Put differently, emotion regulation difficulties may be associated with how one responds to triggering stimuli rather than how often they are exposed to such stimuli. This finding may be useful for clinicians treating misophonia. Specifically, in an effort to reduce difficulties with emotion regulation when treating individuals suffering from misophonia, it may be important to target problems with emotional and behavioral responses to trigger sounds.

These findings add to the emerging literature indicating emotion regulation is relevant to understanding misophonia and has potentially important implications for intervention development [13]. Evidence-based cognitive behavioral therapies that target processes related to emotion regulation are well established for depressive, anxiety, and emotional disorders [31–34]. It is possible that the application of these treatments can help remediate symptoms of misophonia. Although there are no evidence-based treatments specifically for misophonia, an emerging clinical literature suggests the application of a range of interventions within the broader family of cognitive behavioral therapies may be useful when treating misophonia [13, 33]. Without more randomized controlled trials or controlled experimental studies testing interventions for misophonia, it is clear that more research is needed to establish the acceptability and efficacy of any treatment approaches for misophonia.

These results should be considered in the context of the limitations of this study. First, the sample was recruited from Amazon’s MTurk and had a higher percentage of White participants (83.2%) than is representative of the United States population (76.3%; https://www.census.gov/quickfacts/fact/table/US/PST045219), limiting the generalizability of study results. Future studies with diverse and under-represented samples are needed. There may also be associated limitations and concerns regarding data collection and sampling using MTurk, including less sustained attention and fidelity to survey procedures, perhaps in part due to the remote, anonymous nature of study administration. There may also be individual differences among MTurk participants that contribute to limited generalizability of results. For example, MTurk participants scored lower on extraversion and emotional stability than community participants on a measure of the Big Five Personality traits [35] a finding reflected in the current sample’s ALS-18 scores. Although our sample passed all three attention checks throughout the study, and numerous empirical studies have suggested MTurk participants produce reliable results consistent with decision-making biases present in control groups [36], replicability among in-person community samples are warranted.

Second, although the MQ has been used in recent studies [14, 37–40], it was developed and validated using an undergraduate student sample [6]. Limited validity data have been published for the MQ, and its applicability to non-student samples remains unclear. Additionally, impairment on the MQ is assessed by a single item, limiting reliability of this assessment. Third, our data were cross-sectional, liming our ability to draw conclusions about causal relationships between the relevant constructs over time. Future research should aim to collect longitudinal data (e.g., ecological momentary assessment) to further elucidate the relationship between emotions and behaviors in the context of misophonia and emotion regulation strategies. This knowledge can lead to targeted intervention development.

A final limitation to consider is that emotion regulation is a multifaceted construct. For example, Gratz and Roemer [22], who developed the measure on which the DERS-SF is based, proposed emotion regulation includes components such as awareness and understanding of emotions, acceptance of emotions, engaging in goal directed behavior when experiencing strong emotions, inhibiting impulsive behavior, and accessing effective emotion regulation strategies. In this study, we assessed emotion regulation in a global manner, but did not examine the relationships among specific components of emotion regulation with misophonia. The finding indicating a significant relationship between misophonia and emotion regulation even when controlling for other psychological difficulties (e.g., anxiety, depression, COVID-19 impact) highlights the need for future research designed to parse apart components of emotion regulation that contribute to misophonia severity. When considering the small amount of variance accounted for in the present study by difficulties with emotion regulation over and above the impact of COVID-19, anxiety, and depressive symptoms, it is especially important for future studies to discern whether and which specific difficulties with emotion regulation are most germane to understanding misophonia. Further, components of depression and anxiety, assessed in the PHQ and GAD respectively, remained significantly associated with difficulties regulating emotions when misophonia variables were included in regression analyses. This result highlights the challenges associated with exploring the relationship of emotion regulation and misophonia while controlling for other psychopathology that is also characterized by difficulties with emotion regulation. On one hand, the significant relationship of misophonia with difficulties in emotion regulation while controlling for depression and anxiety suggests further exploration of misophonia and emotion regulation is warranted. On the other hand, the significant relationship of all three constructs with emotion regulation raises the potential that the relationship of misophonia with emotion regulation is driven by the fact that people with more severe misophonia symptoms often report more depression and anxiety, but that misophonia is neither uniquely associated with emotion regulation alone nor with symptoms of depression or anxiety alone. One way to disentangle the relationships among depression, anxiety, and emotion regulation in misophonia would be to conduct prospective studies (e.g., ecological momentary assessment) that examine the variability of these phenomena longitudinally.

Despite these limitations, this study is among the first to explore and disentangle the relationships among misophonia and emotional functioning broadly. We hope that these findings will help pave the way for future research to continue examining the role of emotion regulation in the etiology, maintenance, and treatment of misophonia.
